# Supplementary material for: Adaptation to novel spatially-structured environments is driven by the capsule and alters virulence-associated traits
Source: Nat Commun. 2022 Aug 13;13:4751. doi: 10.1038/s41467-022-32504-9 (PMC9376106; doi:10.1038/s41467-022-32504-9)
Supplement: Supplementary file 1 — Supplementary Information [file 41467_2022_32504_MOESM1_ESM.docx]

# **Supplementary Information**

# **Adaptation to novel spatially-structured environments is driven by the capsule and alters virulence-associated traits**

Nucci et al.

# SUPPLEMENTARY FIGURES

**Figure S1. End-point populations are fitter than the ancestor. (A)** Competition between randomly sampled populations and their respective streptomycin-resistant ancestor were performed at an initial 1:1 ratio. Fitness is expressed as the proportion of the evolved population at T_24_/T_0._ Each individual empty point represents an independent competition. Points over 1 indicate the evolved population is fitter than the respective ancestor. Full points represent the mean of the independent experiments. Error bars indicate the standard deviation. **B**. Growth curves of all ancestors and all streptomycin-resistant ancestors in the different media were performed in 96-well microtiter plates, in at least three independent biological replicates. ‘R’ stands for streptomycin-resistant ancestor and ‘S’ stands for streptomycin-sensitive. **C**. Area under the growth curve of each ancestor and the respective streptomycin-resistant clone in the different media. The area was calculated by the function *trapz* from the pracma package for R. All pairwise comparisons between the streptomycin –resistant and streptomycin sensitive ancestors across all environments were statistically, not significantly different (Kruskal Wallis, P> 0.05). For all, error bars indicate standard deviation from the mean. Source data are provided as a Source Data file.

**Figure S2.** **Biofilm formation, surface polysacchairde productoin and population yield of ancestors and evovled clones.** Panels on the left indicate biofilm formation (**A**), surface polysaccharide production (**C**) and population yield expressed as CFU/mL (**E**) of each ancestral clone in each environment. Each dot represents a biologically independent experiment. Panels on the right show biofilm formation (**B**), surface polysaccharide production (**D**) and population yield (**F**) of each population in each environment relative to their respective ancestor. Data for HMP is provided in Figure 5. Values over 1 reflect trait improvement compared to the ancestor. Bars represent within-treament averages (Treatment statistics provided in Table S1), and error bars indicate standard deviation from the mean. Each dot reflects the average of at least three independent biological replicates of each independently evolving population. Individual error bars for each dot are not presented for clarity purposes. Source data are provided as a Source Data file.

Statistics: To test differences of each environment x genotype for each trait compared to the ancestor, we performed One-sample t-tests, and tested the difference from 1. * P < 0.05 ; ** P < 0.01 *** P < 0.001, n.s., not significant. N = number of independently evolved populations analyzed, ie. Number of points on top of bars, (mostly N = 6 independently evolved populations).

To test for differences between capsulated and non-capsulated populations we performed two-sided paired T-tests. Horizontal red bars indicate significant differences between capsulated and their respective non capsulated ancestor. The weight of the red bars indicate different P-values ranging from P < 0.001 to P<0.05. Absence of bar indicates no significant differences. N = 3 independent biological replicates.

**Figure S3. Analysis of mutations in independent clones.** **A**. Number of mutations observed per clone. Data is represented as a boxplot in which the median is highlighted, and dots are outliers. The box represents the upper and lower quantile and whiskers represent 1.5X the interquartile range. No significant differences across groups (Two-way ANOVA, Tukey HSD *post hoc*). **B**. Number of independent clones in which a given gene is mutated. Only genes that are mutated in more than two clones per strain are represented. FAM_XXXX belongs to genes of unknown function, to which a family number was assigned when identifying the pangenome. “IS element” corresponds to an IS missing from its native position. XX…YY corresponds to deletion and XX/YY indicate intergenic mutations between adjacent genes. Source data are provided as a Source Data file.

**Figure S4. Emergence of non-capsulated clones during the evolution experiment.** Different colours represent independently evolving populations. Source data are provided as a Source Data file.


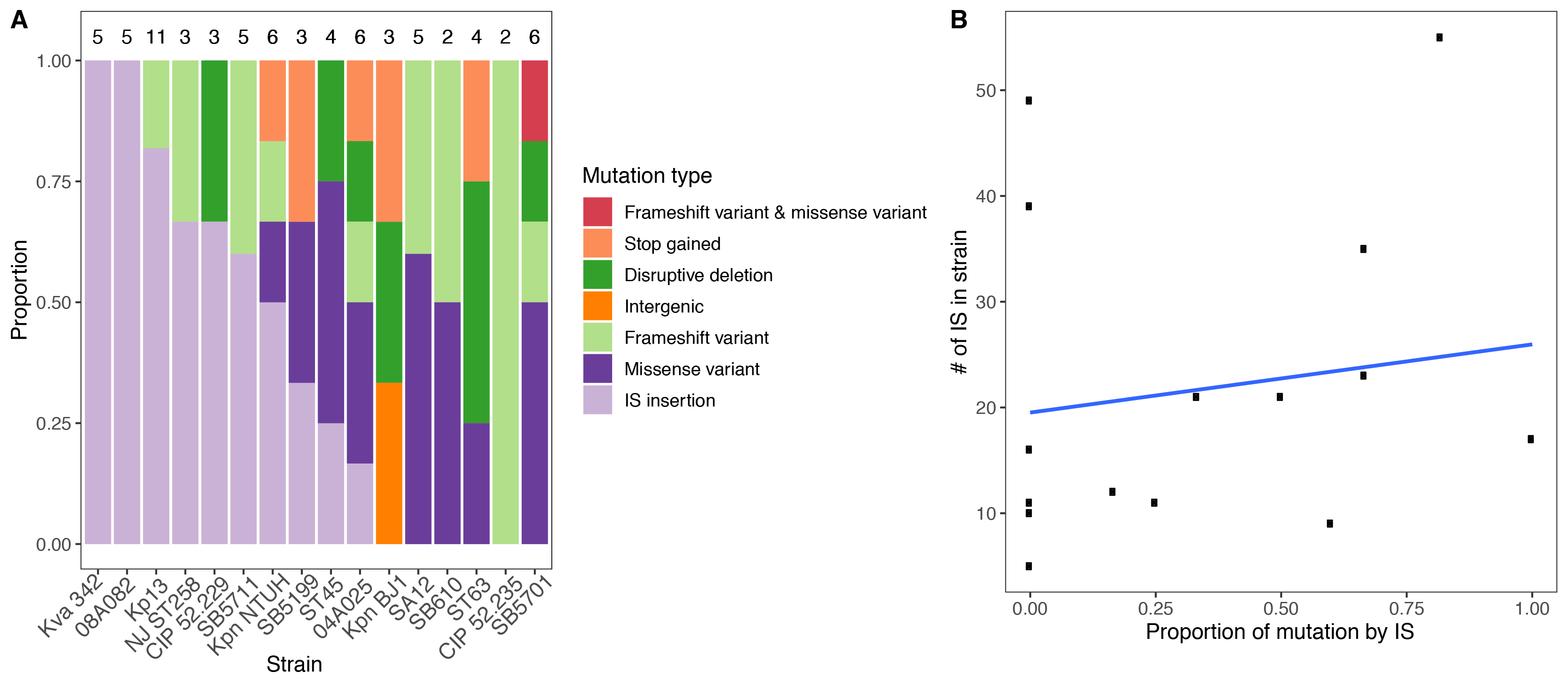


**Figure S5.** **Capsule operon often mutates by IS elements. A**. Proportion of each type of mutation leading to a non-capsulated phenotype in each strain of the short evolution experiment in well-mixed environments. Number on top of bars represent the total number of clones analysed per strain. **B.** Correlation between the number of IS in a strain and the proportion of mutations in the capsule operon caused by IS insertion. Each dot corresponds to one strain. The blue line represents the trend line of a linear model (GLM, P> 0.05). Source data are provided as a Source Data file.

**
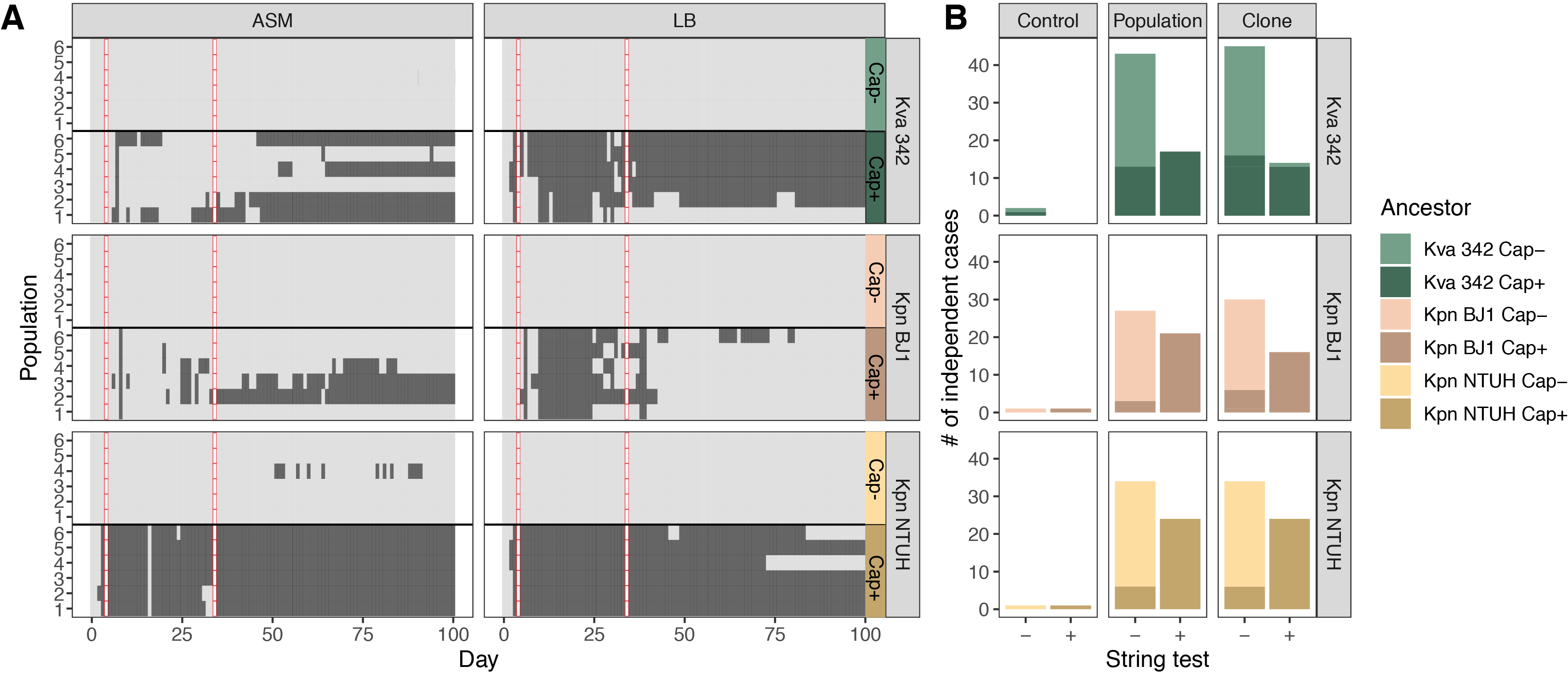
**

**Figure S6. Evolution of HMP during the experiment. A.** Daily observation of HMP in liquid culture. Each line represents and independent population. Black represent HMP, light grey colour represents the ancestral states, and white surrounded by red squares represent absence of recording of HMP phenotype. **B.** Number of clones and populations showing HMP on agar (string-test). Controls correspond to ancestral clones. Source data are provided as a Source Data file.


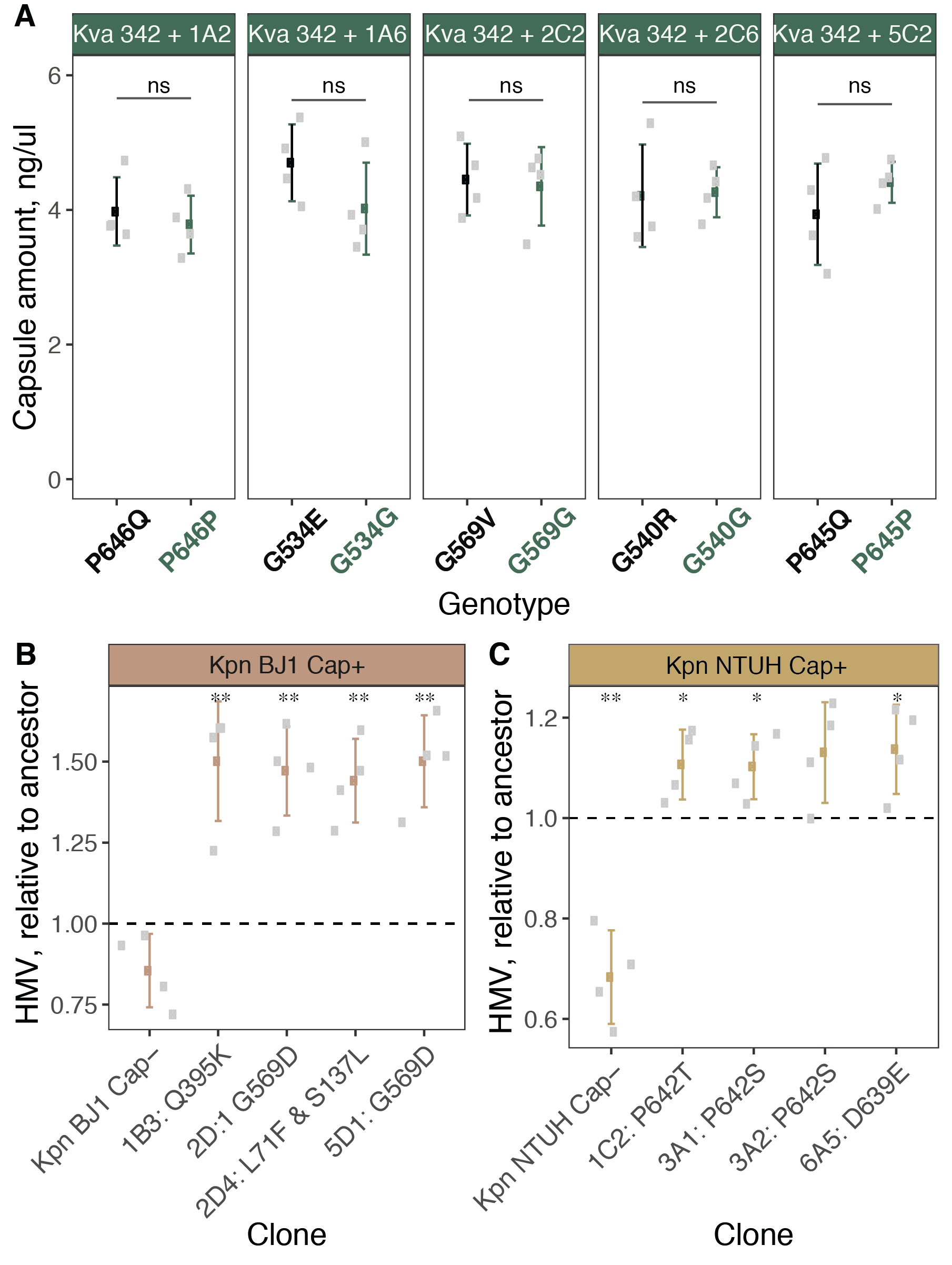


**Figure S7. Characteristics of HMP mutations and clones. A.** Capsule quantification of *wzc* evolved alleles in the ancestral background. Individual dots represent independent experiments, error bars correspond to the standard deviation of the mean. ns not significant, t-student test. **B and C.** HMP of several evolved clones with mutations in *wzc* and evolved in different environments (1B3 and 1C2 in ASM; 2D1, 2D4,3A1 and 3A2 in LB; 5D1 and 6A5 in AUM). HMP is expressed as hypermucoviscosity (HMV) index relative to the capsulated. Non-capsulated ancestor was included as a control. Individual dots represent independent experiments, error bars correspond to the standard deviation of the mean. One-sample t-test, difference from 1. * P < 0.05,** P < 0.01. Source data are provided as a Source Data file.


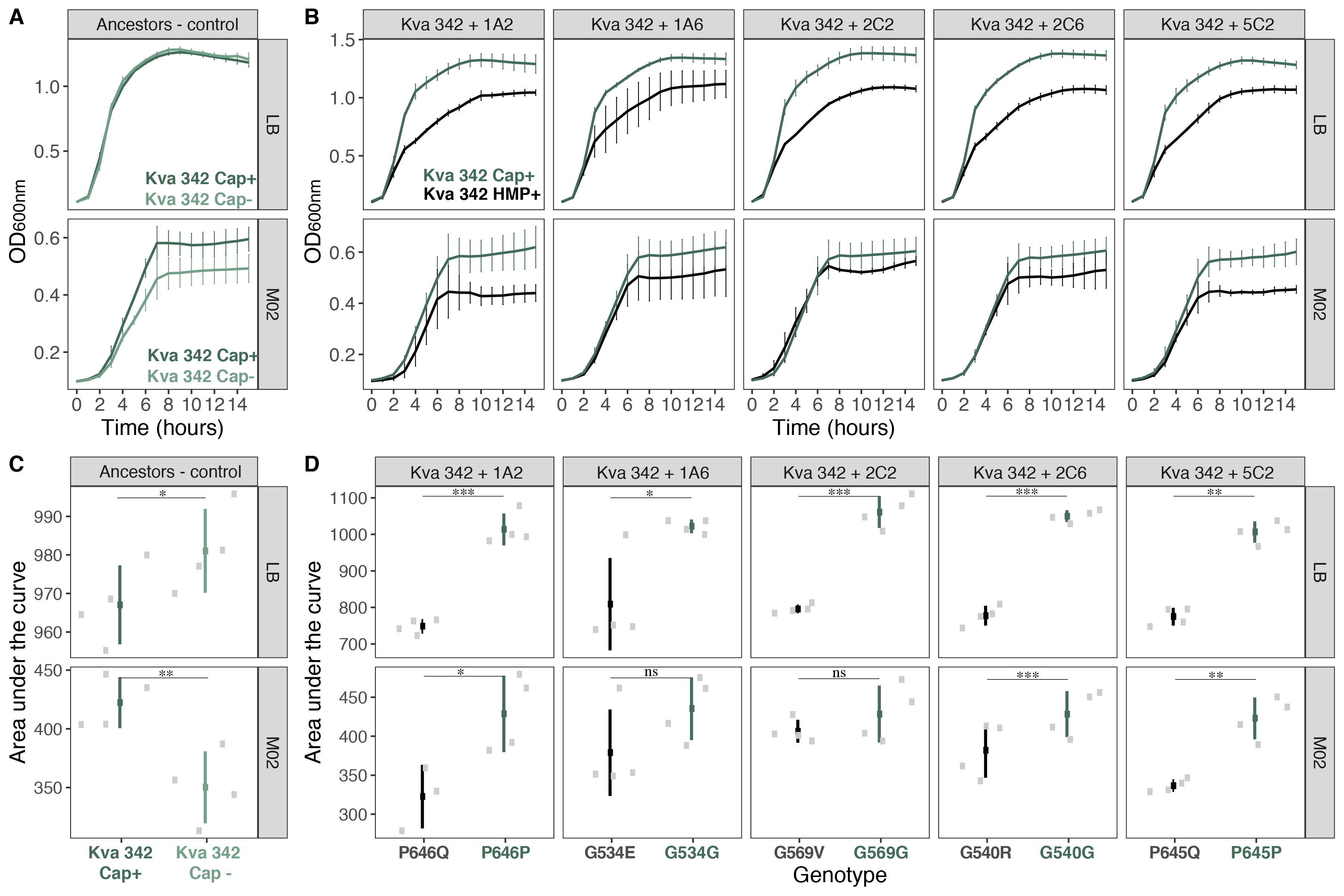


**Figure S8. Cost of HMP during fast growth.** Growth curves (**A** and **B**) and area under the growth curve of capsulated (dark green) and non-capsulated ancestors (light green) (**C** and **D**) of ancestral controls and engineered strains in which the evolved *wzc* allele was reintroduced into the ancestral background (black) and a control clone in which after double recombination event the ancestral allele remained (dark green). Black or green points represent the average, error bars indicated standard deviation from the mean, and grey points indicate each individual biological replicate. Area under the curve was calculated using the *trapz* formula from the *pracma* package for R. Statistics were calculated using two-sided paired t-tests. * P <0.05,** P < 0.01, *** P < 0.001. Source data are provided as a Source Data file.

**Figure S9. Median MIC of selected evolved clones with mutations in *ramA* (A) or in *romA/ramR* (B).** Clone number and the specific mutation are indicated. The median of three independent experiments is depicted. Source data are provided as a Source Data file.

**Figure S10. Growth of *ramA* mutants.** Growth curves of ancestors (green), evolved clones (grey) and engineered strains (ancestral allele in light blue and evolved allele in dark blue). Each panel represents a different evolved clone and mutation. Insertion of evolved allele in a capsulated background (dark blue in panels **B-D**) does not affect growth, but insertion of evolved *ramA* allele in non-capsulated background reduces growth rate (panel **A**), suggesting increased membrane instability in the absence of capsule. The mean and standard deviation (error bars) are indicated and have been derived from three independent biological replicates. Source data are provided as a Source Data file.

# SUPPLEMENTARY TABLES

**Table S1. Median relative differences to the ancestor for each strain in each environment**. For each experiment, all data was transformed by subtracting ancestral values from those of the evolved clones. Numbers in italics indicate that the treatment group is significantly different from the ancestor (One-sample t-test, difference from 0). Cap+ vs Cap- column indicates significant difference between populations evolved from capsulated or from non-capsulated ancestors (t test, * P <0.05; ** P< 0.01; ***P< 0.001). The last column indicates whether capsulated and non-capsulated populations evolved, on average, in the same or opposite direction.

| **Trait** | **Environment** | **Strain** | **Cap+** | **Cap-** | **Cap+ vs Cap-** | **Direction** |
| --- | --- | --- | --- | --- | --- | --- |
| Biofilm | ASM | Kva 342 | *0.54* | *-0.58* | ns | Opposite |
|  |  | Kpn BJ1 | 0.2 | 0.14 | ns | Same |
|  |  | Kpn NTUH | *-1.22* | *0.3* | *** | Opposite |
|  | AUM | Kva 342 | *-0.07* | *-0.07* | ns | Same |
|  |  | Kpn BJ1 | 0 | *0.09* | ns | Same |
|  |  | Kpn NTUH | *0.16* | 0 | ** | Same |
|  | LB | Kva 342 | *1.07* | *0.55* | ns | Same |
|  |  | Kpn BJ1 | *-0.28* | *1.02* | *** | Opposite |
|  |  | Kpn NTUH | *-2.69* | *0.6* | *** | Opposite |
|  | M02 | Kva 342 | *-0.35* | -0.08 | * | Same |
|  |  | Kpn BJ1 | *-0.48* | *0.21* | ** | Opposite |
|  |  | Kpn NTUH | -0.08 | 0 | ns |  |
|  | Soil | Kva 342 | - | - | - |  |
|  |  | Kpn NTUH | - | - | - |  |
| Capsule | ASM | Kva 342 | - | - | - |  |
|  |  | Kpn BJ1 | - | - | - |  |
|  |  | Kpn NTUH | - | - | - |  |
|  | AUM | Kva 342 | 0.33 | *0.08* | ns | Same |
|  |  | Kpn BJ1 | *-1.48* | 0.15 | ** | Opposite |
|  |  | Kpn NTUH | *-3.58* | 0.04 | *** | Opposite |
|  | LB | Kva 342 | *5.97* | *0.46* | * | Same |
|  |  | Kpn BJ1 | -1.52 | *0.65* | * | Opposite |
|  |  | Kpn NTUH | *-6.89* | *0.33* | *** | Opposite |
|  | M02 | Kva 342 | 0.12 | -0.07 | ns | Opposite |
|  |  | Kpn BJ1 | 0.08 | *-0.09* | ns | Opposite |
|  |  | Kpn NTUH | *-1.42* | -0.01 | *** | Same |
|  | Soil | Kva 342 | - | - | - |  |
|  |  | Kpn NTUH | - | - | - |  |
| CFU | ASM | Kva 342 | - | *0.31* | - |  |
|  |  | Kpn BJ1 | - | 0.08 | - |  |
|  |  | Kpn NTUH | - | *0.35* | - |  |
|  | AUM | Kva 342 | 0.05 | *0.25* | * | Same |
|  |  | Kpn BJ1 | *0.27* | *0.26* | ns | Same |
|  |  | Kpn NTUH | -0.05 | *-0.05* | ns | Same |
|  | LB | Kva 342 | - | *0.44* | - |  |
|  |  | Kpn BJ1 | - | *0.42* | - |  |
|  |  | Kpn NTUH | - | *0.28* | - |  |
|  | M02 | Kva 342 | *0.07* | 0.08 | ns | Same |
|  |  | Kpn BJ1 | -0.02 | *-0.19* | *** | Same |
|  |  | Kpn NTUH | *-0.15* | 0.01 | *** | Opposite |
|  | Soil | Kva 342 | *0.22* | -0.01 | *** | Opposite |
|  |  | Kpn NTUH | *0.1* | 0.02 | *** | Same |
| HMP | ASM | Kva 342 | *0.54* | *1.54* | * | Same |
|  |  | Kpn BJ1 | 0.28 | *0.36* | ns | Same |
|  |  | Kpn NTUH | *0.11* | 0.03 | ns | Same |
|  | AUM | Kva 342 | *0.81* | *2.48* | ** | Same |
|  |  | Kpn BJ1 | *0.72* | *0.5* | ns | Same |
|  |  | Kpn NTUH | -0.08 | 0 | ns |  |
|  | LB | Kva 342 | *0.66* | *2.64* | ** | Same |
|  |  | Kpn BJ1 | *0.66* | -0.16 | ns | Opposite |
|  |  | Kpn NTUH | 0.02 | 0.08 | ns | Same |
|  | M02 | Kva 342 | *0.97* | *1.48* | ns | Same |
|  |  | Kpn BJ1 | *0.27* | 0.13 | * | Same |
|  |  | Kpn NTUH | *-0.18* | *0.68* | ** | Opposite |
|  | Soil | Kva 342 | *0.39* | *1.69* | *** | Same |
|  |  | Kpn NTUH | -0.03 | *0.69* | *** | Opposite |

**Table S2. Statistical analyses of trait evolution. A.** Statistical analyses of multifactorial ANOVA for all traits analysed. dF stands for degrees of freedom.  **B.** Bidirectional stepwise regression. ‘+’ represents the order in which the variables were introduced. AIC is the Akaike information criterion.

**A.**

| **Trait** | **Variable** | **dF** | **F value** | **P value** |
| --- | --- | --- | --- | --- |
| Capsule production | Strain | 2 | 62.25 | 5.75e-12 |
|  | Capsule genotype | 1 | 13.01 | 0.0005 |
|  | Environment | 3 | 1.45 | 0.23 |
| Yield (CFU) | Strain | 2 | 10.98 | 0.0012 |
|  | Capsule genotype | 1 | 19.32 | 2.52e-05 |
|  | Environment | 4 | 29.8 | < 2e-16 |
| Biofilm formation | Strain | 2 | 86.25 | 6.30e-16 |
|  | Capsule genotype | 1 | 73.24 | 3.51e-14 |
|  | Environment | 3 | 3.14 | 0.03 |
| HMP | Strain | 2 | 63.06 | 5.20e-13 |
|  | Capsule genotype | 1 | 21.67 | 7.29e-06 |
|  | Environment | 4 | 1.304 | 0.27 |

**B.**

| **Trait** | **Step** | **dF** | **AIC** |
| --- | --- | --- | --- |
| Biofilm formation |  |  | -76.11 |
|  | + Capsule genotype | -1 | -86.95 |
|  | + Strain | -2 | -98.74 |
| Yield (CFU) |  |  | -390.56 |
|  | + Environment | -4 | -462.05 |
|  | + Strain | -2 | -470.13 |
| HMP |  |  | -67.20 |
|  | + Strain | -2 | -166.67 |
|  | + Capsule genotype | -1 | -194.80 |
| Capsule production |  |  | 195.89 |
|  | + Strain | -2 | 170.09 |
|  | + Capsule genotype | -1 | 165.64 |

**Table S3. Dissimilarity matrix of mutated genes across ancestors.** In italics, the dissimilarities in terms of mutations at the gene level between capsulated and non-capsulated variants from the same ancestor are highlighted. The matrix was calculated using the R package diverse, and the function dis_categories, with the cosine method.

|  | **Kpn BJ1 Cap+** | **Kpn BJ1 Cap -** | **Kpn NTUH Cap+** | **Kpn NTUH Cap-** | **Kva 342 Cap+** | **Kva 342 Cap-** |
| --- | --- | --- | --- | --- | --- | --- |
| **Kpn BJ1 Cap+** | 0.00 |  |  |  |  |  |
| **Kpn BJ1 Cap -** | *0.36* | 0.00 |  |  |  |  |
| **Kpn NTUH Cap+** | 0.82 | 0.98 | 0.00 |  |  |  |
| **Kpn NTUH Cap-** | 0.93 | 0.84 | *0.7* | 0.00 |  |  |
| **Kva 342 Cap+** | 0.79 | 0.88 | 0.68 | 0.84 | 0.00 |  |
| **Kva 342 Cap-** | 0.94 | 0.87 | 0.96 | 0.83 | *0.67* | 0.00 |

**Table S4.** **List of mutations in non-capsulated clones descending from capsulated clones from the evolution experiment**. bp, base pair; SNP, single nucleotide polymorphism; pos, position; * premature stop codon.

| Clone ID | Strain | Environment | Gene | Mutation type | Change |
| --- | --- | --- | --- | --- | --- |
| 1A3 | Kva 342 | ASM | *wcaJ* | IS insertion | pos: 188 |
| 1A5 | Kva 342 | ASM | *wcaJ* | IS insertion | pos: 1342 |
| 2C1 | Kva 342 | LB | *wcaJ* | IS insertion | pos: 1204 |
| 1B1 | Kpn BJ1 | ASM | *wcaJ* | non-synonymous | R393Q |
| 1B4 | Kpn BJ1 | ASM | *wcaJ* | 1 bp deletion | pos: 261 |
| 1B6 | Kpn BJ1 | ASM | *wcaJ* | non-synonymous | K327E |
| 2D2 | Kpn BJ1 | LB | *wcaJ* | 1 bp deletion | pos: 126 (stretch of 7 As) |
| 2D2 | Kpn BJ1 | LB | *wzc* | non-synonymous | Q389R |
| 2D3 | Kpn BJ1 | LB | *wcaJ* | 1 bp deletion | pos: 126 (stretch of 7 As) |
| 4C1 | Kpn NTUH | M02 | *wcaJ* | IS insertion | pos: 1066 |
| 4C3 | Kpn NTUH | M02 | *wzc* | 1 bp insertion | pos: 118 |
| 4C3 | Kpn NTUH | M02 | *rmpA/hyp_prot* | 1 bp deletion | intergenic |
| 4C4 | Kpn NTUH | M02 | *rfaH* | 1 bp insertion | pos: 107 |
| 7C1 | Kpn NTUH | Soil | *rfaH* | non-synonymous | Q18* |

**Table S5.** **N/S ratio.** A 2:1 transition to transversion ratio is assumed. Calculations were performed using gdtools package and following *breseq* documentation. Bases that are covered by UN (unknown) and MASK (masked) regions by *breseq* were not taken into account in this analysis. To calculate whether the observed is significantly different to the expected, a two-sided binomial test was performed. For strain Kpn BJ1, taking into account the heterozygous plasmid present in the strain in the analyses, did not alter qualitatively the result.

| Model | Strain | N | S | N/S observed | N/S expected | P value |
| --- | --- | --- | --- | --- | --- | --- |
| Permissive | Kva 432 | 156 | 31 | 5.032 | 2.272 | P < 0.001 |
|  | Kpn BJ1 | 49 | 56 | 0.875 | 2.269 | P < 0.001 |
|  | Kpn NTUH | 58 | 11 | 5.273 | 2.269 | P = 0.008 |
| Conservative | Kva 432 | 156 | 31 | 5.032 | 2 | P < 0.001 |
|  | Kpn BJ1 | 49 | 56 | 0.875 | 2.016 | P < 0.001 |
|  | Kpn NTUH | 58 | 11 | 5.273 | 2.354 | P=0.01 |

**Table S6. Mutations in *wzc* gene in Kva 342 clones found to be string-test positive.**

| Clone ID | Environment | Gene | Mutation type | Change |
| --- | --- | --- | --- | --- |
| 1A1 | ASM | *wzc* | Non-synonymous | D643H |
| 1A2 | ASM | *wzc* | Non-synonymous | P646Q |
| 1A4 | ASM | *wzc* | Non-synonymous | D643E |
| 1A6 | ASM | *wzc* | Non-synonymous | G534E |
| 2C2 | LB | *wzc* | Non-synonymous | G569V |
| 2C3 | LB | *wzc* | Non-synonymous | G540E |
| 2C4 | LB | *wzc* | Non-synonymous | G540E |
| 2C5 | LB | *wzc* | Non-synonymous | G540E |
| 2C6 | LB | *wzc* | Non-synonymous | G540R |
| 5C2 | AUM | *wzc* | Non-synonymous | P645Q |

**Table S7. Mutations in *romA*/*ramR* /*ramA* and its regulon.**

| Environment | Pop # | Ancestral strain | ID | Position | Change | Mutation | Annotation | Description |
| --- | --- | --- | --- | --- | --- | --- | --- | --- |
| ASM | 2 | Kva 342 Cap+ | 1A2 | 4086331 | G→C | H31D (CAT→GAT) | *ramA* | Regulatory protein SoxS |
| ASM | 3 | Kva 342 Cap+ | 1A3 | 1180139 | C→T | R41W (CGG→TGG) | *rob* | Right origin‑binding protein |
| ASM | 3 | Kva 342 Cap+ | 1A3 | 4086318 | G→A | S35L (TCG→TTG) | *ramA* | Regulatory protein SoxS |
| ASM | 4 | Kva 342 Cap+ | 1A4 | 4086247 | T→A | K59* (AAG→TAG) | *ramA* | Regulatory protein SoxS |
| ASM | 4 | Kva 342 Cap+ | 1A4 | 1179806 | IS insertion | intergenic (‑118/‑213) | *bepF/rob* | Efflux pump periplasmic linker BepF/Right origin‑binding protein |
| ASM | 5 | Kva 342 Cap+ | 1A5 | 4277409 | Δ15 bp | coding (3064‑3078/3147 nt) | *acrB* | Multidrug efflux pump subunit AcrB |
| ASM | 6 | Kva 342 Cap+ | 1A6 | 1179519 | Δ9240 | Δ9240 | *[bepF]–[uvrD2]* | 9-gene deletion including *rob* |
| ASM | 4 | Kva 342 Cap- | 1D4 | 4276402 | +A | coding (2057/3147 nt) | *acrB* | Multidrug efflux pump subunit AcrB |
| ASM | 6 | Kva 342 Cap- | 1D6 | 4087438 | G→A | Q39* (CAG→TAG) | *romA* | hypothetical protein |
| LB | 1 | Kva 342 Cap+ | 2C1 | 4058089 | Δ33295 | Δ33295 | *tpa–HJIKBMFB_03868* | Large deletion of 68 genes including *ramA,romA,ramR* |
| LB | 2 | Kva 342 Cap+ | 2C2 | 4086201 | A→G | V74A (GTC→GCC) | *ramA* | Regulatory protein SoxS |
| LB | 3 | Kva 342 Cap+ | 2C3 | 4087318 | C→A | E79* (GAA→TAA) | *romA* | hypothetical protein |
| LB | 4 | Kva 342 Cap+ | 2C4 | 4087318 | C→A | E79* (GAA→TAA) | *romA* | hypothetical protein |
| LB | 5 | Kva 342 Cap+ | 2C5 | 4087318 | C→A | E79* (GAA→TAA) | *romA* | hypothetical protein |
| LB | 6 | Kva 342 Cap+ | 2C6 | 4275301 | C→A | S319* (TCG→TAG) | *acrB* | Multidrug efflux pump subunit AcrB |
| LB | 1 | Kva 342 Cap- | 3B1 | 4087308 | Δ867 bp | Δ867 bp | *[romA]–[ramR]* | [romA], [ramR] |
| M02 | 3 | Kva 342 Cap- | 4D3 | 4086201 | A→G | V74A (GTC→GCC) | *ramA* | Regulatory protein SoxS |
| M02 | 1 | Kpn BJ1 Cap- | 5A1 | 1733268 | Δ1 bp | coding (387/1194 nt) | *acrA* | Multidrug efflux pump subunit AcrA |
| M02 | 4 | Kpn BJ1 Cap- | 5A4 | 4514441 | Δ7 bp | coding (196‑202/366 nt) | *rob* | Right origin‑binding protein |
| AUM | 3 | Kva 342 Cap+ | 5C3 | 4086589 | IS insertion | coding (964/1101 nt) | *romA* | hypothetical protein |
| AUM | 4 | Kva 342 Cap+ | 5C4 | 4086460 | A→C | W365G (TGG→GGG) | *romA* | hypothetical protein |
| AUM | 5 | Kva 342 Cap+ | 5C5 | 4086342 | (GATAT)1→2 | coding (80/342 nt) | *ramA* | Regulatory protein SoxS,superoxide response regulon |
| AUM | 6 | Kva 342 Cap+ | 5C6 | 4087438 | G→A | Q39* (CAG→TAG) | *romA* | hypothetical protein |
| AUM | 1 | Kva 342 Cap- | 6B1 | 4087218 | C→T | W112* (TGG→TAG) | *romA* | hypothetical protein |
| AUM | 2 | Kva 342 Cap- | 6B2 | 4086150 | C→T | R91H (CGC→CAC) | *ramA* | Regulatory protein SoxS |
| AUM | 3 | Kva 342 Cap- | 6B3 | 4086352 | G→A | R24C (CGT→TGT) | *ramA* | Regulatory protein SoxS |
| AUM | 4 | Kva 342 Cap- | 6B4 | 4086303 | +T | coding (119/342 nt) | *ramA* | Regulatory protein SoxS |
| AUM | 5 | Kva 342 Cap- | 6B5 | 4086123 | G→A | P100L (CCG→CTG) | *ramA* | Regulatory protein SoxS |
| AUM | 6 | Kva 342 Cap- | 6B6 | 4086311 | C→A | W37C (TGG→TGT) | *ramA* | Regulatory protein SoxS |
| Soil | 1 | Kva 342 Cap+ | 7A1 | 1179823 | IS insertion | intergenic (‑135/‑196) | *bepF/rob* | Efflux pump periplasmic linker BepF/Right origin‑binding protein |
| Soil | 2 | Kva 342 Cap+ | 7A2 | 1180251 | C→T | A78V (GCG→GTG) | *rob* | Right origin‑binding protein |
| Soil | 2 | Kva 342 Cap+ | 7A2 | 4078449 | Δ74,528 bp | Δ74,528 bp | *[bepG]–[cadB]* | Large deletion of 68 genes including *ramA,romA,ramR* |
| Soil | 3 | Kva 342 Cap+ | 7A3 | 1180127 | T→A | W37R (TGG→AGG) | *rob* | Right origin‑binding protein |
| Soil | 3 | Kva 342 Cap+ | 7A3 | 4087438 | G→A | Q39* (CAG→TAG) | *romA* | hypothetical protein |
| Soil | 4 | Kva 342 Cap+ | 7A4 | 4086318 | G→T | S35* (TCG→TAG) | *ramA* | Regulatory protein SoxS |
| Soil | 1 | Kva 342 Cap+ | 7D1 | 4058821 | Δ32830 bp | Δ32830 bp | *tpa–HJIKBMFB_03868* | Large deletion including *ramA,romA,ramR* |
| Soil | 5 | Kva 342 Cap- | 7D5 | 1179851 | Δ1 bp | intergenic (‑163/‑168) | *bepF / rob* | Efflux pump periplasmic linker BepF/Right origin‑binding protein |
| Soil | 6 | Kva 342 Cap- | 7D6 | 4087438 | G→A | Q39* (CAG→TAG) | *romA* | hypothetical protein |

**Table S8. Primers used in this study.**

| **Primer name** | **Sequence** | | **Sense** | | | **Construction** |
| --- | --- | --- | --- | --- | --- | --- |
| **wcaJ complementation** | | | | | | |
| pUCP28T.gibson.5 | CTCTAGAGGATCCCCGGGTAC | | Forward | | | Amplification of expression vector pUCP28T |
| pUCP28T.gibson.3 | TCGACCTGCAGGCATGCAAGC | | Reverse | | | Amplification of expression vector pUCP28T |
| 56_pUCP28T _wcaJ_3 | gttgtaaaacgacggccagtgccaagCTTGCATGCCTGCAGGTCCTTATCTTAAGCAGCATCTTA | | Reverse | | | Cloning of *wcaJ* from Kpn NTUH in plasmid pUCP28T |
| 56_pUCP28T_wcaJ_5 | CCATGATTACGAATTCGAGCTCGGTACCCGGGGATCCTCTAGAGGGTTCAATATTTTAAGGAAATCTTC | | Forward | | | Cloning of *wcaJ* from Kpn NTUH in plasmid pUCP28T |
| 26_pUCP28T_wcaJ_5 | CCATGATTACGAATTCGAGCTCGGTACCCGGGGATCCTCTAGAG | | Forward | | | Cloning of *wcaJ* from Kpn BJ1 in plasmid pUCP28T |
| 26_pUCP28T_wcaJ_3 | gttgtaaaacgacggccagtgccaagCTTGCATGCCTGCAGGTCccttgtcttaaggagcatctaa | | Reverse | | | Cloning of *wcaJ* from Kpn BJ1 in plasmid pUCP28T |
| 24_pUCP28T_wcaJ_5 | CCATGATTACGAATTCGAGCTCGGTACCCGGGGATCCTCTAGAGgaatttacaaaagagtaaataact | | Forward | | | Cloning of *wcaJ* from Kva 342 in plasmid pUCP28T |
| 24_pUCP28T_wcaJ_3 | gttgtaaaacgacggccagtgccaagCTTGCATGCCTGCAGGTCaactttaagcgcattacaaat | | Reverse | | | Cloning of *wcaJ* from Kva 342 in plasmid pUCP28T |
| pUCP28T.verif5 | GGGCCTCTTCGCTATTACG | | Forward | | | Verification of insert in expression vector pUCP28T |
| pUCP28T.verif3 | AATACGCAAACCGCCTCTC | | Reverse | | | Verification of insert in expression vector pUCP28T |
| **Construction of wzc mutants** | | |  | |  | |
| 24_Wzc_pKNGgibson-5v2 | CATAAGTAGAAGCAGCAACCCAAGTAGCTTTACCAGCATCggaatagaatcgcctgaagaactcg | | Forward | | | Cloning of *wzc* from Kva 342 in plasmid pKNG101 |
| 24_Wzc_pKNGgibson-3v3 | CTTCCGCTCAGGTCCTTGTCCTTTAACGAGGATTGTTACgccaccctgcattagaattac | | Reverse | | | Cloning of *wzc* from Kva 342 in plasmid pKNG101 |
| **Construction of ramA mutants** | | | | | | |
| ramA.24. pKNGGibson5 | cataagtagaagcagcaacccaagtagctttaccagcatcATGACGATTTCCGCTCAGG | | Forward | | | Cloning of *ramA* from Kva 342 in plasmid pKNG101 |
| ramA.24. pKNGGibson3 | cttccgctcaggtccttgtcctttaacgaggattgttacAACGGTAAACCAGGAGAGC | | Reverse | | | Cloning of *ramA* from Kva 342 in plasmid pKNG101 |
| ramA.24.seq5 | ATGAAACGGCTCAGGCTGC | | Forward | | | Sanger sequencing of *ramA* |
| ramA.24.seq3 | CTCTCCTGTCTGCTGTTGC | | Reverse | | | Sanger sequencing of *ramA* |
| **pknG101 primers** | | | | | | |
| pkNG101.gibson.5 | GTAACAATCCTCGTTAAAGGAC | | Forward | Amplification of pKNG101 vector | | |
| pkNG101.gibson.3 | GATGCTGGTAAAGCTACTTG | | Reverse | Amplification of pKNG101 vector | | |
| pknG101.verif5 | CTACATATCACAACGTGCGTGG | | Forward | Verification of insert in pKNG101 vector | | |
| pknG101.verif3 | | ACCAAGCCTATGCCTACAGC | Reverse | Verification of insert in pKNG101 vector | | |
| **Specific wza primers** |  | |  |  | | |
| KL30.wza.5 | CGAAAGTCCTCAGGTTGATGT | | Forward | Verification of KL30 (Kva 342) capsule | | |
| KL30.wza.3 | GACCTCCAGCGGCATTTAT | | Reverse | Verification of KL30 (Kva 342) capsule | | |
| KL2.wza.5 | GCATTAGCATTGGCGATTGG | | Forward | Verification of KL2 (Kpn BJ1) capsule | | |
| KL2.wza.3 | AAGCTCCACCACGTTCTTAC | | Reverse | Verification of KL2 (Kpn BJ1) capsule | | |
| KL1.wza.5 | GGATCACCCGGAATTGACTAC | | Forward | Verification of KL1(Kpn NTUH) capsule | | |
| KL1.wza.3 | TTCCAGCTACTTGCACCTTAC | | Reverse | Verification of KL1 (KPN NTUH) capsule | | |
