# Supplementary material for: Adaptation to novel spatially-structured environments is driven by the capsule and alters virulence-associated traits
Source: Nat Commun. 2022 Aug 13;13:4751. doi: 10.1038/s41467-022-32504-9 (PMC9376106; doi:10.1038/s41467-022-32504-9)
Supplement: Supplementary file 3 — Description of Additional Supplementary Files [file 41467_2022_32504_MOESM3_ESM.pdf]

Description of Additional Supplementary Files

File Name: Supplementary Data 1
Description: All tables related with the bioinformatics analyses, details of each evolving population as well as the analyses of whole genome sequencing.
