# Supplementary material for: Adaptation to novel spatially-structured environments is driven by the capsule and alters virulence-associated traits
Source: Nat Commun. 2022 Aug 13;13:4751. doi: 10.1038/s41467-022-32504-9 (PMC9376106; doi:10.1038/s41467-022-32504-9)
Supplement: Supplementary file 5 — Reporting Summary [file 41467_2022_32504_MOESM5_ESM.pdf]

nature portfolio

Corresponding author(s): Olaya Rendueles
Last updated by author(s): Jul 4, 2022

Reporting Summary

Nature Portfolio wishes to improve the reproducibility of the work that we publish. This form provides structure for consistency and transparency in reporting. For further information on Nature Portfolio policies, see our Editorial Policies and the Editorial Policy Checklist.

Statistics

For all statistical analyses, confirm that the following items are present in the figure legend, table legend, main text, or Methods section.

n/a	Confirmed	
☐	☒	The exact sample size (*n*) for each experimental group/condition, given as a discrete number and unit of measurement
☐	☒	A statement on whether measurements were taken from distinct samples or whether the same sample was measured repeatedly
☐	☒	The statistical test(s) used AND whether they are one- or two-sided<br>*Only common tests should be described solely by name; describe more complex techniques in the Methods section.*
☐	☒	A description of all covariates tested
☐	☒	A description of any assumptions or corrections, such as tests of normality and adjustment for multiple comparisons
☐	☒	A full description of the statistical parameters including central tendency (e.g. means) or other basic estimates (e.g. regression coefficient) AND variation (e.g. standard deviation) or associated estimates of uncertainty (e.g. confidence intervals)
☐	☒	For null hypothesis testing, the test statistic (e.g. *F*, *t*, *r*) with confidence intervals, effect sizes, degrees of freedom and *P* value noted<br>*Give P values as exact values whenever suitable.*
☒	☐	For Bayesian analysis, information on the choice of priors and Markov chain Monte Carlo settings
☒	☐	For hierarchical and complex designs, identification of the appropriate level for tests and full reporting of outcomes
☒	☐	Estimates of effect sizes (e.g. Cohen's *d*, Pearson's *r*), indicating how they were calculated

Our web collection on statistics for biologists contains articles on many of the points above.

Software and code

Policy information about availability of computer code

Data collection	all data was analysed using open source and/or free software. We used Panacota v1.0, prokka v1.12 for annotation,Kleborate v1.0 for Klebsiella analyses, ISFinder downloaded on January 2021, breseq 0.26v1 and Snippy, gdtools package v0.3, BlastP v2.7.1.+. COG data was downloaded via NCBI's FTP site (ftp://ftp.ncbi.nlm.nih.gov/pub/COG/COG2014/data/, March 2021) and we used the provided python script merger.py to generate the COG database.
Data analysis	The data was analyzed with R version 3.5.3 and Rstudio version 1.2. We used tidyverse v 1.3.1, dplyr v0.8.3, drawProteins v1.2, pracma v2.3.3, stringr v1.4. Graphs were generated using ggplot v3.3.5, cowplot v1.1.1.

For manuscripts utilizing custom algorithms or software that are central to the research but not yet described in published literature, software must be made available to editors and reviewers. We strongly encourage code deposition in a community repository (e.g. GitHub). See the Nature Portfolio guidelines for submitting code & software for further information.

Data

Policy information about availability of data

All manuscripts must include a data availability statement. This statement should provide the following information, where applicable:

- Accession codes, unique identifiers, or web links for publicly available datasets
- A description of any restrictions on data availability
- For clinical datasets or third party data, please ensure that the statement adheres to our policy

All data generated in this study have been deposited in the public repository Figshare (ref 92 in manuscript) under accession code https://figshare.com/articles/

dataset/RawData_Nuccietal_2022/19597195. The processed data generated in this study are provided in the Supplementary Information/Source Data file. Dataset S1 includes all tables related with the bioinformatics analyses, details of each evolving population as well as the analyses of whole genome sequencing.

Human research participants

Policy information about studies involving human research participants and Sex and Gender in Research.

Reporting on sex and gender	n/a
Population characteristics	n/a
Recruitment	n/a
Ethics oversight	n/a

Note that full information on the approval of the study protocol must also be provided in the manuscript.

Field-specific reporting

Please select the one below that is the best fit for your research. If you are not sure, read the appropriate sections before making your selection.

- [x] Life sciences
- [ ] Behavioural & social sciences
- [ ] Ecological, evolutionary & environmental sciences

For a reference copy of the document with all sections, see nature.com/documents/nr-reporting-summary-flat.pdf

Life sciences study design

All studies must disclose on these points even when the disclosure is negative.

Sample size	All independently evolved populations were examined, thus when comparing the effect of environment x genotype against its ancestor, N most commonly = 6 for each treatment. The 95% confidence limits in terms of multiples of sigma rapidly tighten until n reaches 6, thus, we decided for each treatment to have n=6 populations
Data exclusions	Four populations out of the 168 were found to be contaminated and were excluded from all further analyses, as described in the manuscript.
Replication	For the evolution experiment, each independently evolving culture from a genotype x environment, was initiated from a single culture. was initiated by a single culture. Quantification of capsulated clones throughout the experiment and determination of their hypermucoviscosity (Figure S4 and S6) was determined once, as the experiment was running. All other experimental procedures were performed in three biologically independent replicates. Technical replicates were performed, but the mean of each technical replicate was taken into account for statistical purposes.
Randomization	Each experimental group (genotype x environment) consisted of six independently evolving populations.<br>To perform contamination tests throughout the evolution experiment, we used randomization which was carried out by the "sample" function in R. For all other tests, randomnization is not applicable, as all populations were tested for each phenotype and each genotype analyzed and compared to its respective ancestor in its evolutionary environment.
Blinding	Blinding is not applicable to its study. Each population was tested in its evolutionary environment against its respective ancestor.

Reporting for specific materials, systems and methods

We require information from authors about some types of materials, experimental systems and methods used in many studies. Here, indicate whether each material, system or method listed is relevant to your study. If you are not sure if a list item applies to your research, read the appropriate section before selecting a response.

Materials & experimental systems

n/a	Involved in the study
x	Antibodies
x	Eukaryotic cell lines
x	Palaeontology and archaeology
	x Animals and other organisms
x	Clinical data
x	Dual use research of concern

Methods

n/a	Involved in the study
x	ChIP-seq
x	Flow cytometry
x	MRI-based neuroimaging

Animals and other research organisms

Policy information about studies involving animals; ARRIVE guidelines recommended for reporting animal research, and Sex and Gender in Research

Laboratory animals	n/a
Wild animals	n/a
Reporting on sex	n/a
Field-collected samples	We used three bacterial strains: two Klebsiella pneumoniae and one Klebsiella variicola. All of which have been collected by other laboratories, and the appropriate references are provided in the text.
Ethics oversight	n/a

Note that full information on the approval of the study protocol must also be provided in the manuscript.
